# The clinical nurse specialist's role in head and neck cancer care: United Kingdom National Multidisciplinary Guidelines

**DOI:** 10.1017/S0022215116000657

**Published:** 2016-05

**Authors:** L Dempsey, S Orr, S Lane, A Scott

**Affiliations:** 1 Aintree University Hospitals NHS Foundation Trust, Liverpool, UK; 2 University College Hospitals London (UCLH), British Association of Head and Neck Oncology Nurses, UK; 3 East & North Hertfordshire NHS Trust, British Association of Head and Neck Oncology Nurses, UK

## Abstract

This is the official guideline endorsed by the specialty associations involved in the care of head and neck cancer patients in the UK. It discusses the role of the clinical nurse specialist in the head and neck cancer patient journey and provides recommendations on the clinical nurse specialist led assessments and interventions for this group of patients receiving cancer care.

**Recommendations:**

• All cancer patients should meet a clinical nurse specialist at the point of diagnosis. (R)

• Clinical nurse specialists must act as gate keeper to the patients' cancer pathway to provide a seamless journey. (R)

• Holistic needs assessment should be completed at different stages of the patient's pathway to reflect the changes of the patients' needs. (R)

• Clinical nurse specialists to be part of local and national initiatives for health promotion and raising awareness in the public domain. (G)

• Clinical nurse specialists should lead in redesigning of services and policies to ensure they are responsive to patient's needs for the future. (G)

• Treatment summaries should become part of practice to provide good communication between primary and secondary care to enable continuity of care for the patient. (G)

## Introduction

Distress is common among cancer patients; it is multi-factorial, comprising of psychological, social and spiritual elements which can impact on an individual's ability to cope effectively with cancer. A diagnosis of cancer leaves patients frightened and vulnerable, often unable to understand the full implication of the treatment they are being offered.^1^ It can, in extreme cases, impact on the ability to adhere to treatment and self-management. It is widely recognised that head and neck cancer patients are particularly vulnerable to psychological distress as many suffer life-changing, long-term consequences resulting from the cancer and the treatment.^2^^,^^3^

Carers of patients with head and neck cancer are under considerable stress during and after treatment as a result of disruption to daily life, the financial and emotional strain of long-term treatment and in many cases, role reversal within the family unit. Patients and carers look to healthcare professionals to provide information to help manage the psychological and social elements of head and neck cancer.^4^ Supportive care, appropriate information and individualised care planning is a key to improve the experience of the patient and the carer.

It is the function of the clinical nurse specialist (CNS) to give the patient and carer the wherewithal to cope with the diagnosis, treatment and long-term consequences through the use of empathy and experience.^5^^–^^8^ The Cancer Reform Strategy^9^ recognises that the CNS is critical in the delivery of information, communication and co-ordination of care. It has been recognised that care co-ordination individualised to the patient during and after treatment is vital to deliver appropriate person-centred care.^10^ The CNS' role within the multidisciplinary team (MDT) also allows for easy and timely referral on to other resources, i.e. palliative care and psychological support.

## The role of the clinical nurse specialist (figure 1)

The National Institute for Health and Care Excellence Improving Outcomes Guidance^11^ identified the key worker as ‘A person who with the patient's consent and agreement takes a key role in co-ordinating the patient's care and promoting continuity, ensuring the patient knows who to access for information and advice’. The CNS will act as the patient's key worker during their cancer pathway by providing specialist cancer knowledge and expertise to both the patient and carer, which can be both complex and disjointed, involving interventions from multiple professionals or agencies.^6^ The CNS reinforces and imparts their specialist knowledge to the other professionals and agencies to improve the cancer process and in turn will improve the cancer journey for the patient and carer. The CNS may pass the key worker role on to another relevant professional when the patient is on a particular part of the pathway, as this may be in their best interests and provide the best support at that particular point of their journey.

**Fig. 1:**
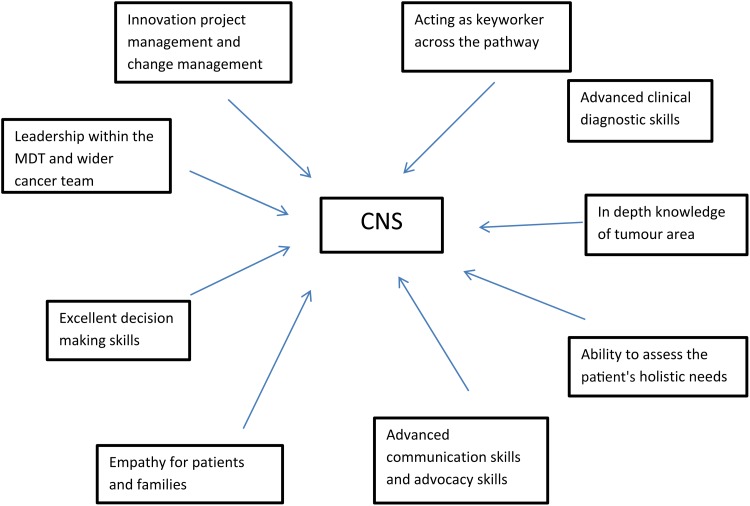
Key contributions of the clinical nurse specialist to cancer care.

The CNS workload can be complex and varied dependent on the patient's needs, it can be categorised into themes:

• Specialist technical knowledge of the cancer process and treatment options
• Acting as the patient’s key worker for a specific part of the process and linking in with the MDT
• Utilising advanced communication skills to support the patient and carer psychologically through the disease process
• Lead on redesigning services to make them responsive to the patient's needs
• Health education and promotion to reduce the risk of recurrence and promote a healthy lifestyle
• Assisting in local and national initiatives to promote awareness and prevention.

## The role of the CNS within the MDT

The CNS acts as the key accessible professional to the patient within this multiprofessional setting, and allows the CNS to influence the patient's pathway. They are well placed to support the patient at each stage of their pathway and promote integration within the team. The CNS should be recognised as the patient's advocate within the MDT meetings where they deliver patient-centred care tailored to the individual patient's needs. In acting as the patients' advocate, the CNS also plays a key role in ensuring that the multidisciplinary care is responsive to the patients' needs and preferences.^12^

## The CNS and the patient's pathway

Clinical nurse specialists increasingly take the lead role in shaping patient care pathways and refining systems to make a difference to the patient experience and their safety. By acting as the key worker,^13^ they provide information, support and liaison to improve the cancer care process for the patient. They can track the stage of their pathway and ensure it is seamless and prevent any problems from occurring. They are well placed with in the organisation to assist in system changes to ensure the pathway represents a quality service that fulfils the standards of cancer care and patients' expectations.

## The patient's role as advocate

Patients and carers have always been at the centre of cancer services, but have not always been encouraged or empowered to help influence and shape them.^14^ Patient support groups have traditionally played a large part in providing information, companionship and peer support for patients and carers throughout their cancer journey. They can also influence policy and services at local, national and at international level.

The Cancer Plan^15^ saw the patient involvement being embedded in cancer services. This involvement has continued with organisations like National Cancer Research Institute (NCRI) having representation on all its clinical study groups and funding committees. This is also happening on cancer strategy committees and clinical network groups.

Patients and carers now have a realisation that they have considerable influence in gaining access to treatments and medicines, can participate in the designing of clinical trials and influence the amount of money spent by central government on research.^14^ It is important to understand that patients provide a very different perspective on benefits *vs* risks of treatment.^16^ They will very often opt for extensive and often life threatening treatment even if the benefits and outcomes are unclear.

## Managing patients' and carers' expectations

A diagnosis of cancer has far reaching effects beyond the patient to their loved ones. This life-changing experience means that relationships and roles and responsibilities can often be changed. Treatments for head and neck cancer can have devastating effects on the lives of patients, including disfiguration, speech and swallowing impairment.^17^

‘The Recovery Package’^18^ has been designed by Macmillan Cancer Support to help ‘provide a series of key interventions, which when delivered together can greatly improve outcomes for people living with and beyond cancer’. It is made up of:

• Holistic needs assessment
• Treatment summary
• Cancer care review
• Education and support events.

It also compliments stratified care plans, which enable individualised follow-up care and self-support. It facilitates urgent access back to the specialist team if needed or on-going support from healthcare professionals.

**Recommendations**

• All cancer patients should meet a cinical nurse specialist at the point of diagnosis (R)
• Clinical nurse specialists must act as gate keeper to the patient's cancer pathway to provide a seamless journey (R)
• Holistic needs assessment (HNA) should be completed at different stages of the patient's pathway to reflect the changes of the patients' needs (R)
• Clinical nurse specialists to be part of local and national initiatives for health promotion and raising awareness in the public domain (G)
• Clinical nurse specialists should lead in redesigning of services and policies to ensure they are responsive to patient's needs for the future (G)
• Treatment summaries should become part of practice to provide good communication between primary and secondary care to enable continuity of care for the patient (G)

### Holistic needs assessment

An HNA ensures that the patients' and carers' physical, emotional and social needs are met in a timely and appropriate way, and that advice and support is available from the right source at the right time.^19^ The HNA is the process of assessing the patient and/or carers by developing an understanding of what the person with cancer understands and needs at diagnosis and various time points thereafter which can be agreed by the MDT or when clinically appropriate (i.e. disease progression). This discussion may cover all or some of the following areas – physical, spiritual, emotional, social and environmental needs. Undertaking holistic needs assessment with a patient enables them to more fully engage in their own care and make informed choices. The information gathered at the HNA can also be shared with other members of the MDT and also have influence on service needs and data collection. The National Cancer Survivorship Initiative (NCSI) in 2010 highlights HNA as one of its ‘Key Shifts’ as well as it being a Peer Review Measure.

Different assessment tools are used, i.e. distress thermometer, concerns checklist and more recently the patient concerns inventory. The tools can be used at different stages along the patient trajectory, but with an emphasis being on assessing and eliciting patients' and carers' concerns and expectations. This leads to a discussion and care planning of the patients' needs and helps to manage expectations.

Pre-treatment clinics provide an opportunity for patients and carers to meet the CNS and other allied health professionals prior to surgical or oncological treatment. It allows HNA assessment to take place, but also facilitates discussion of acute treatment and rehabilitation with the key professionals involved prior to commencement of such.

### Care plans

A care plan is based on the diagnosis and holistic assessment of the patient.^19^ It will highlight the patient's issues, outlining any actions, approaches and timings to address them. Care plans change during the patient pathway according to the patient's needs at any one time; however, any change should be discussed and actions agreed with the patient. By working with the patient to develop care plans following an HNA, the patient is able to take more control of what happens to them and support themselves to self-manage their condition.^20^

### Treatment summary

A key component to effective patient care is good communication between the primary and secondary care sectors. Making sure that general practitioners are fully informed about their patients’ cancer journeys can ease the transition between acute and long-term care. For this to be effective, treatment summaries are provided at the end of any acute treatment by the MDT for the general practitioner and patient. The treatment summary describes the treatment that that person has received, including any adverse reactions, the side effects and signs and symptoms of recurrence. This treatment summary provides confidence to the patient that their care is continuing albeit in the community setting. Patients report feelings of abandonment and vulnerability once initial treatment is complete but with a treatment summary and care plan these feelings can be minimised. Patients' on-going self-management can be well supported through peer support groups and health and wellbeing events. The CNS is obliged to inform patients of what is available in the local area that may be accessed by the patient and carer.

### Key points

Clinical nurse specialists should:

• meet every patient at the point of diagnosis to assist in a smooth transition along the cancer pathway
• ensure effective communication within the MDT, with patient and carer and within the community setting
• be at the centre of the patient's pathway and make effective use of resources
• act as the patient advocate, utilising support groups to act as patient voices in the changing healthcare environment to make them patient-centred
• perform holistic needs assessment for all patients at diagnosis and specific points along their journey to ensure patient-focused care is being provided
• offer treatment summaries to all people involved in the patient's recovery to ensure effective communication
• offer individualised care plans to help patients take control of the recovery phase.
